# Assessing the Link Between Physical Activity and Musculoskeletal Disorders in Taxi Drivers: A Comparison of Accelerometry and Self‐Report Measures

**DOI:** 10.1002/msc.70201

**Published:** 2026-03-06

**Authors:** Marta Marín‐Berges, Pablo A. Lizana, Isabel Iguacel, Marcos Echevarría‐Polo, Valentina Marroquín‐Pinochet, Constanza Rivas‐Sanhueza, German Vicente‐Rodríguez, Alejandro Gómez‐Bruton

**Affiliations:** ^1^ IBiOPS Instituto de Investigación Sanitaria Aragón Universidad de Zaragoza Zaragoza Spain; ^2^ Departamento de Fisiatría y Enfermería, Facultad de Ciencias de la Salud Universidad de Zaragoza Zaragoza Spain; ^3^ Laboratory of Epidemiology and Morphological Sciences Instituto de Biología Pontificia Universidad Católica de Valparaíso Valparaíso Chile; ^4^ Center for Interdisciplinary Research in Biomedicine, Biotechnology and Well‐Being (CID3B) Pontificia Universidad Católica de Valparaíso Valparaíso Chile; ^5^ Instituto Agroalimentario de Aragón (IA2) Zaragoza Spain; ^6^ NUTRI‐GENUD (Growth, Exercise, Nutrition and Development), Faculty of Health Sciences University of Zaragoza Zaragoza Spain; ^7^ Centro de Investigación Biomédica en Red de Fisiopatología de la Obesidad y Nutrición (CIBEROBN) Instituto de Salud Carlos III Madrid Spain; ^8^ EXER‐GENUD (Growth, Exercise, Nutrition and Development), Faculty of Health and Sport Sciences University of Zaragoza Huesca Spain; ^9^ Facultad de Ciencias de la Salud y del Deporte, Departamento de fisiatría y enfermería Universidad de Zaragoza Zaragoza Spain

**Keywords:** GT9X link accelerometer, IPAQ, musculoskeletal disorders, physical activity, taxi drivers

## Abstract

**Background:**

Taxi drivers are at an elevated risk for work‐related musculoskeletal disorders (MSDs), and the reasons for this are prolonged sitting, static postures, and whole‐body vibration. Physical activity (PA) may mitigate MSD risk but is often assessed subjectively. This study compared objective (wrist‐worn accelerometry) and subjective (IPAQ‐S) measures of PA and sedentary behaviours by examining their associations with MSD prevalence in taxi drivers from Spain and Chile.

**Methods:**

In this cross‐sectional study, 170 taxi drivers (mean age 51.9 ± 10.7 years; 87.1% male) completed sociodemographic and Nordic questionnaires on MSDs (7‐day recall), and the IPAQ‐S for PA. A subsample of 36 wore a wrist‐worn accelerometer for seven days to quantify sedentary time and PA amount and intensities.

**Results:**

Overall, 68.8% of drivers reported pain in at least one body region in the past 7 days, most commonly the neck (36.5%) and lower back (32.9%). IPAQ‐S overestimated moderate‐to‐vigorous PA (145.7 ± 140.2 vs. 42.4 ± 31.2 min/day, *p* < 0.01) compared with accelerometry, with moderate correlation (*r* = 0.47). Sedentary time averaged 715.5 ± 146.9 min/day by accelerometry, with negligible correlation to IPAQ‐S (*r* = −0.01). Female drivers had higher odds of neck, upper back, and lower back pain, while higher BMI was associated with knee pain. No significant associations were found between PA levels and MSD prevalence.

**Conclusions:**

Taxi drivers exhibit high MSD prevalence, extreme sedentary exposure, and marked overestimation of PA in self‐reports. Neither self‐reported nor accelerometer‐measured physical activity was significantly associated with 7‐day MSD prevalence. Targeted interventions should combine ergonomic improvements, active breaks, and accurate monitoring tools to reduce occupational health risks.

## 1 Introduction

Musculoskeletal disorders (MSDs) are defined as alterations in body structures such as muscles, joints, tendons, ligaments, nerves, cartilage, bones, and the local circulatory system (Isusi 2020). When they are caused or aggravated mainly by work and the effects of the immediate environment in which it is carried out, they are called work‐related MSDs (Isusi 2020).

MSDs are a global public health problem that negatively affects quality of life, causes incapacity and, in the long term, disability (Woolf and Pfleger 2003). They are the leading cause of years lived with disability (YLD) worldwide, accounting for an estimated 149 million YLD, representing 17% of the total global burden of non‐fatal health loss (World Health Organization 2022).

These disorders affect millions of workers in various occupations, especially those that involve a fast pace of work, repetitive movement patterns, insufficient recovery time, heavy lifting, non‐neutral body postures or vibration on the body, high demands or low control, among others (Isusi 2020; Punnett and Wegman 2004).

Professional drivers are considered a high‐risk occupational group for MSDs, with prevalence rates ranging from 43.1% to 93.0%, most commonly affecting the lower back (Joseph et al. 2020). A systematic review and meta‐analysis involving 5277 taxi drivers confirmed that the lower back was the most frequently affected area (53.9%), followed by the neck (38.2%), shoulders (34.9%), upper back (18.3%), and knees (14.1%) (Rezaei et al. 2024).

When using the Standardised Nordic Questionnaire (SNQ) with a 12‐month recall period in taxi drivers, we found a high prevalence of musculoskeletal disorders (MSDs) (83.0%), with the lumbar region (51.6%), neck (50.2%), and right shoulder (34.5%) being the most commonly affected areas. Furthermore, significant associations were observed between multisite pain and poorer physical quality of life, particularly among older drivers and those reporting perceived financial instability (Marín‐Berges et al. 2026). However, additional relevant variables that may influence the development of MSDs, such as sedentary behaviours or physical activity, could not be properly analysed due to inconsistencies in the data collection timeframes. In most studies, the Standardised Nordic Questionnaire (SNQ) captures MSD‐related data over a 12‐month period, whereas physical activity is typically assessed using weekly self‐reports or accelerometry, making temporal comparisons difficult. Despite this limitation, substantial evidence supports the protective role of moderate to high levels of physical activity (300–450 min per week) in reducing musculoskeletal pain across various body regions (Rhim et al. 2022). Conversely, sedentary behaviour—particularly prolonged sitting—has emerged as a major risk factor for MSDs in professional drivers (Murray et al. 2019), and is now considered a global pandemic in industrialised societies (Cunningham et al. 2020; Kohl et al. 2012). Notably, replacing just 1 hour of sedentary behaviour with physical activity may lower the risk of low back pain by up to 8% (Zhang et al. 2024).

In addition, low levels of physical activity are associated with a higher Body Mass Index (BMI), which in turn has been identified as a contributing factor to the development and exacerbation of MSDs in the lower back (Shiri et al. 2010). High BMI increases biomechanical loading and systemic inflammation, both of which are linked to increased incidence and severity of musculoskeletal pain (Vincent et al. 2013).

Recent estimates indicate that nearly one‐third of the global adult population, approximately 1.8 billion people, are physically inactive (Strain et al. 2024). This widespread inactivity contributes significantly to the global burden of chronic non‐communicable diseases, including obesity, type 2 diabetes, hypertension, cardiovascular disease, and even premature mortality (Manson et al. 2004).

Therefore, accurately assessing physical activity and inactivity levels in taxi drivers is essential. However, few studies have specifically addressed this issue. For example, a study conducted in Cameroon found that 62.9% of drivers reported low levels of physical activity (Ngatcha Tchounga et al. 2022). Similarly, in Colombia, 36.4% of drivers engaged only in low‐intensity physical activity (Melo Betancourt et al. 2021). In another investigation, nearly 40% of drivers stated they had not performed any moderate physical activity in the previous month, and 63.9% reported no vigorous activity at all (Mirpuri et al. 2021). However, none of them assessed the link to MSDs.

Although these figures may seem inconsistent, the disparity could be attributed to the subjective nature of self‐reported data and the use of tools not validated for use with taxi drivers, a population characterised by prolonged sitting and, in many cases, having little time for exercise. Additionally, cross‐study differences may reflect country‐specific factors such as variations in urban infrastructure, work conditions, cultural attitudes toward physical activity, and access to recreational spaces, all of which can influence the activity patterns of taxi drivers. Therefore, further research across diverse national contexts is needed to better understand physical activity behaviours and health‐related implications in this occupational group and to inform targeted interventions.

The objectives of this research were: (1) to describe the level of physical activity and sedentary behaviour of drivers using both objective (accelerometry), and subjective (self‐reported short‐form International Physical Activity Questionnaire, IPAQ‐S) measures; (2) to evaluate the association between the prevalence of musculoskeletal disorders (MSDs) and the levels of physical activity and sedentary behaviour in this population; and (3) to analyse the discrepancies between assessment methods (objective vs. subjective) and discuss their implications for research and public health practice.

## 2 Methodology

### 2.1 Study Design and Participants

This was a cross‐sectional observational study. The sampling process employed a combination of non‐probability convenience and snowball sampling methods to recruit participants. This dual approach allowed researchers to initially select participants from known networks and then expand the sample by encouraging participants to recommend other taxi drivers. Data collection took place over 1 year, from October 2022 to November 2023, ensuring comprehensive capture of health data across different seasons and periods of the year, which may have different effects on taxi drivers’ health conditions.

The inclusion criteria for this study were set to focus on active taxi drivers working in the cities of Zaragoza, Spain, or Valparaíso, Chile. Specifically, participants had to meet the following requirements: (1) be actively working as taxi drivers; and (2) work within the city of Zaragoza or the region of Valparaíso. To ensure that drivers had sufficient exposure to the occupational risks and demands of the profession, two exclusion criteria were defined: (1) being disabled at the time of data collection, which would disqualify those unable to work, and (2) having less than 1 year of experience as a taxi driver, given that the study focused on the long‐term health effects of this activity.

### 2.2 Sample Size

An a priori sample size was estimated to obtain acceptable precision for the prevalence of 7‐day musculoskeletal symptoms. We used the single‐proportion formula *n* = *Z* ^2^ × *p*(1 − *p*)/*d* ^2^, assuming 95% confidence (*Z* = 1.96). As no taxi‐driver–specific estimates were available, we considered prevalence figures reported in working populations in Chile (49.8%; Vidal Gamboa et al. 2016) and Spain (40.26%; Ministerio de Sanidad 2021)as a plausible range and selected a conservative expected prevalence of *p* = 0.50 to avoid underestimation. With an absolute precision of *d* = 0.08, the minimum required sample size was 151 participants. We inflated this by 10% to account for incomplete responses, yielding a target of ≥ 167 participants.

### 2.3 Ethics Committee

This study was approved by the Research Ethics Committee of the Autonomous Community of Aragon (PI22‐382) and by the Bioethics Committee of the Pontifical Catholic University of Valparaíso (No. BIOEPUCV‐H 633‐2023). All participants gave their voluntary consent by signing the informed consent form after being fully informed about the objectives of the study. They were also informed of their right to withdraw from the study at any time without consequences.

### 2.4 Recruitment of Participants

Before beginning the research, the taxi cooperatives in both cities were contacted to request permission to use their facilities for the study. The cooperatives also helped inform taxi drivers about the location, date, and time of the meeting through internal communications, including WhatsApp messages, social media platforms, and their internal magazine. Taxi drivers interested in participating in the study arrived at the cooperative’s facilities and completed the questionnaires online or in person. Two researchers on site addressed any questions or concerns.

### 2.5 Instruments

For this study, four instruments were selected for analysis: an ad hoc questionnaire developed to obtain sociodemographic, personal and occupational information; the SNQ and the IPAQ‐S. As the IPAQ‐S assesses physical activity over the past 7 days, items from that refer to the same recall period that were used to ensure consistency. Additionally, in a randomly selected subsample of 36 participants from the total sample of 170, physical activity was objectively measured using the GT9X Link accelerometer.

Ad Hoc Questionnaire: This questionnaire gathered information on a range of demographic, socioeconomic, and occupational variables, including country, age, gender, marital status, educational level, hours of work per day, years worked as a taxi driver and self‐reported height and weight. BMI was calculated as weight in kilogrammes divided by the square of height in metres (BMI = kg/m^2^).

The SNQ developed by Kuorinka et al., provides information on the prevalence of pain in 15 body regions: neck, right shoulder, left shoulder, right elbow/forearm, left elbow/forearm, right wrist/hand, left wrist/hand, thoracic spine, lumbar spine, right hip/leg, left hip/leg, right knee, left knee, right ankle/foot, and left ankle/foot (Kuorinka et al. 1987). In this case, the subsection for the last 7 days was used. SNQ has been validated for the Spanish and Chilean populations (Martínez and Alvarado Muñoz 2017; Mateos‐González et al. 2024). The Spanish version has shown very high internal consistency (*ω* = 0.81) and excellent test–retest reliability (ICC = 0.95; Mateos‐González et al. 2024). The Chilean version has also demonstrated test–retest reliability for the 7‐day section with moderate‐to‐substantial agreement (*κ* ≈ 0.51–0.85; Martínez and Alvarado Muñoz 2017).

The IPAQ‐S consists of seven questions that provide information on energy expenditure and the time the person spends doing moderate and vigorous physical activity, as well as the time the person spends walking or sitting in the last 7 days (Craig et al. 2003). Data are expressed in MET minutes per week, and sitting time is recorded in hours per day. From the activity variables, two composite measures were derived: moderate‐vigorous physical activity (MVPA), calculated as the sum of time spent on moderate and vigorous activities, and total physical activity, calculated as the sum of time spent walking, on moderate and vigorous activities. This short version has been validated in Spanish (Cancela et al. 2019) and the Chilean population (Palma‐Leal et al. 2022). The original 12‐country evaluation reported high test–retest reliability (Spearman's *ρ* ∼ 0.8; Craig et al. 2003). Evidence supporting construct validity of the Spanish version has also been reported, although self‐report may overestimate activity levels (Cancela et al. 2019). In Chilean samples, test–retest reliability for the IPAQ‐S has been reported with ICC values ranging approximately from 0.56 to 0.89 (Palma‐Leal et al. 2022).

Responses were converted into Metabolic Equivalent Task minutes per week (MET‐min/week), following the IPAQ scoring protocol. The MET values assigned were: 3.3 METs for walking, 4.0 METs for moderate physical activity, and 8.0 METs for vigorous physical activity. The total weekly physical activity score (in MET‐min/week) was calculated by summing the MET‐min/week for walking, moderate, and vigorous activities. Participants were then classified into three activity levels according to IPAQ guidelines:

Inactive: No activity or not enough to meet the minimum criteria.

Minimally active: Meeting at least the equivalent of 600 MET‐min/week of physical activity.

Health‐Enhancing Physical Activity (HEPA): Meeting one of the following: Vigorous activity ≥ 3 days/week totalling ≥ 1500 MET‐min/week, or ≥ 7 days of any combination of walking, moderate or vigorous activity totalling ≥ 3000 MET‐min/week.

An objective measurement of physical activity was obtained using the GT9X Link device (ActiGraph, Pensacola, FL, USA), a validated accelerometer and one of the most widely used devices for measuring physical activity (Suau et al. 2024). This is a compact and lightweight triaxial device (3.5 × 3.5 × 1 cm; 14 g) that detects acceleration along the *X*, *Y*, and *Z* axes (sample rate: 30–100 Hz; dynamic range: ± 8 g). Following the methodology used in previous studies with professional drivers (Gilson et al. 2021), participants wore the device on their non‐dominant wrist. The sensor captured acceleration over 7 consecutive days at 30 Hz with a dynamic range of ± 8 g.

The accelerometry data were processed using the GGIR package (3.1–5 version; Migueles et al. 2019), through the RStudio software (2024.09.1+394 version), which operates on the R programming environment (4.3.2 version). Acceleration signals were processed as proposed by van Hees et al. (2014) for free‐living physical activity assessment.

Non‐wear time was identified as consecutive stationary episodes ≥ 60 min in which the three axes had a standard deviation < 13.0 mg (van Hees et al. 2011). We excluded people who didn’t wear it long enough to support the imputation (< 72 h of wear time or no usage data in each 1 h of the 24‐h cycle).

To determine the time subjects spent at each intensity level (min/day), we used the cut‐off points (acceleration; mg) validated by Hildebrand et al. (2017, 2014) for ActiGraph devices in the adult population worn on the non‐dominant wrist: 44.8 mg for light‐intensity physical activity (LPA), 100.6 mg for moderate‐intensity physical activity (MPA), and 428.8 mg for vigorous‐intensity physical activity (VPA). We quantified the intensity at each defined level in 1‐min periods in which the mean acceleration was higher than the threshold to reduce the likelihood of misclassifying artefacts as activity. Inactivity was also classified when the intensity was below the LPA threshold, MVPA was classified as the sum of MPA and VPA, and total physical activity (TPA) was classified as the sum of LPA, MPA, and VPA.

We also calculated the overall mean acceleration (mg/day), a validated surrogate for global physical activity (van Hees et al. 2011; White et al. 2016). Based on this data, we applied the formula proposed by White et al. (2019, 2016) (−10.58 + 1.1176*x + 2.9418 × sqrt(*x*) − 0.00059277 × (*x*^2)) to obtain the activity in units of MET/min/week.

### 2.6 Statistical Analysis

Descriptive statistics were performed to characterise the sample in terms of sociodemographic, occupational and anthropometric variables. Continuous variables were presented as mean ± standard deviation (SD). Categorical variables were expressed as absolute frequencies and percentages.

To compare physical activity levels and sedentary behaviours between self‐reporting methods (IPAQ‐S) and objective measurements (accelerometry), a subsample of 36 participants with valid accelerometry data was used. The normality of the variables was checked using the Shapiro–Wilk test. Since most variables did not follow a normal distribution, Wilcoxon signed‐rank tests were applied to compare the differences between the two methods for each variable (inactivity/sedentary behaviour, light, moderate, vigorous activity, MVPA and total physical activity).

To assess the association between the variables measured by both instruments, Spearman's correlation coefficients (*ρ*) were calculated. Correlation strength was interpreted following conventional thresholds: *ρ* = 0.00–0.19 (very weak), 0.20–0.39 (weak), 0.40–0.59 (moderate), 0.60–0.79 (strong), and 0.80–1.0 (very strong) (Pavan Kumar and Reddi 2023).

Likewise, logistic regression models were performed to analyse the association between MSDs in different body regions (dichotomous variable: presence/absence in the last 7 days) and sociodemographic variables. Additionally, contingency tables and chi‐square tests were used to explore the association between the presence of MSDs in each body region (dichotomous variable: yes/no in the past 7 days) and variables related to physical activity and sedentary behaviour.

For the self‐reported IPAQ‐S data, the following variables were analyzed: compliance with WHO aerobic recommendations, physical activity classification (Inactive, Minimally Active, HEPA Active), and sedentary time. For the accelerometry‐based data, WHO guidelines compliance and sedentary time were also examined. Compliance with WHO guidelines was defined as engaging in at least 150 min per week of aerobic moderate‐intensity physical activity, or 75 min of vigorous‐intensity activity, or an equivalent combination of both, as recommended for adults aged 18–64 years (Bull et al. 2020).

Sedentary time was dichotomised using a cut‐off point of 650 min/day. This threshold was based on a large harmonised meta‐analysis on accelerometer‐measured physical activity and all‐cause mortality, which identified ≥ 650 min/day as the upper quartile (Q4) of sedentary time (Ekelund et al. 2019). Given the nature of taxi drivers’ occupation, characterised by prolonged sitting, this highest quartile was considered an appropriate reference for defining high sedentary exposure in the present study.

The level of statistical significance was set at *p* < 0.05. All analyses were performed using the Jamovi software version 2.6.44.

## 3 Results

Table 1 shows the sociodemographic characteristics of taxi drivers in both cities. The sample consisted of 170 taxi drivers, with a mean age of 51.90 ± 10.66 years. Most participants were men (87.10%) who worked more than 9 h a day (87.10%) and had an average of 16.50 ± 10.40 years of experience as drivers. The average BMI was 28.5 ± 5.10 kg/m^2^.

**TABLE 1:** Sociodemographic characteristics.

	Total (*n* = 170) (%)
Country Spain/Chile	94 (55.30)/76 (44.70)
Age (mean and SD)	51.90 ± 10.66
Gender male/Female	148 (87.10)/22 (12.90)
Civil status	
Single	44 (25.90)
In a relationship/married	101 (57.40)
Divorced	25 (14.70)
Education^a^	
Primary	27 (16.10)
Secondary	55 (32.70)
Post‐secondary	86 (51.20)
Hours of work per day	
≤ 4 h	1 (0.60)
5–8 h	21 (12.40)
≥ 9 h	148 (87.10)
Years worked as a taxi driver (mean and SD)	16.50 ± 10.35
BMI (mean and SD)^b^	28.50 ± 5.10

Abbreviations: BMI, Body Mass Index; SD, Standard Deviation.

^a^ Missing data for 2 participants.

^b^ Missing data for 1 participant.

**FIGURE 1:**
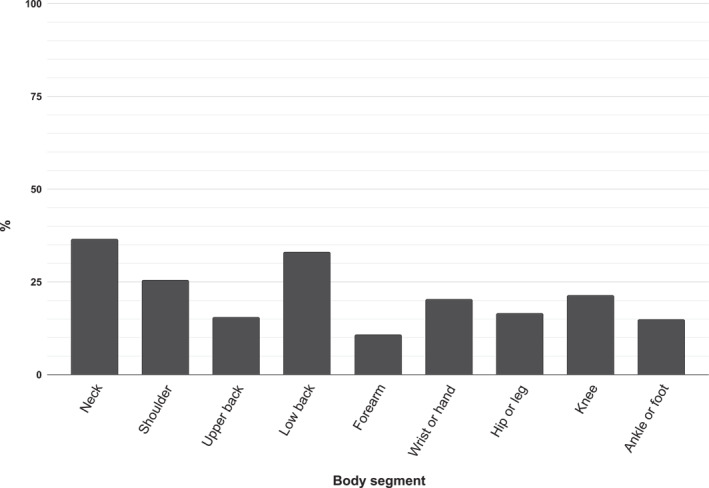
Prevalence of MSD in the past 7 days. Distribution of musculoskeletal disorders reported by taxi drivers during the week prior to the survey. Percentages indicate the proportion of participants affected in each anatomical region: neck (36.50%), lower back (32.90%), upper back (15.30%), shoulders (25.30%), knee (21.20%), hip/thigh (16.50%), feet (14.70%), hands (14.70%), and elbows (10.60%). Overall, 68.8% of participants reported pain in at least one body region.

Figure 1 shows the prevalence of MSDs in the past 7 days. 68.80% of the taxi drivers reported experiencing pain in at least one body region during the past 7 days. The most frequently affected areas were the neck (36.50%), lower back (32.90%), upper back (15.30%), and shoulders (25.30%), followed by the knee (21.20%) and hip/thigh (16.50%). Other areas such as the feet (14.70%), hands (14.70%), and elbows (10.60%) also showed significant levels of involvement.

As shown in Table 2, data collected using the IPAQ‐S showed a high level of sedentary time (622.64 ± 184.47 min/day) and a weekly energy expenditure of 728.70 ± 986.29 MET‐min/week for walking, 636.21 ± 1098.00 MET‐min/week for moderate activity, and 990.26 ± 1709.53 MET‐min/week for vigorous activity, for a combined total of 2355.17 ± 2733.74 MET‐min/week. According to the IPAQ classification, 52.9% of participants were classified as HEPA active (i.e., engaging in health‐enhancing physical activity), 33.5% as minimally active, and 13.5% as inactive.

**TABLE 2:** Physical activity levels and sedentary time according to the IPAQ‐S questionnaire (*n* = 170).

	Mean ± SD	Median (IQR)
Sitting minutes/day	622.64 ± 184.47	600.00 (480.00–720.00)
Walking MET‐minutes/week	728.70 ± 986.29	321.75 (66.00–990.00)
Moderate MET‐minutes/week	636.21 ± 1098.00	120.00 (0.00–720.00)
Vigorous MET‐minutes/week	990.26 ± 1709.53	0.00 (0.00–1440.00)
Moderate to vigorous MET‐minutes/week	1626.47 ± 2339.47	520.00 (0.00–15,120)
Total physical activity MET‐min/week	2355.17 ± 2733.74	1350.00 (390.38–3438.00)
IPAQ‐S classification		
1. Inactive	23 (13.5%)	
2. Minimally active	57 (33.5%)	
3. HEPA active	90 (52.9%)	

Abbreviations: HEPA, Health‐Enhancing Physical Activity; IPAQ‐S, International Physical Activity Questionnaire—Short Form; IQR, Interquartile Range; MET, Metabolic Equivalent of Task; SD, Standard Deviation.

From the subsample of 36 drivers who carried the GT9X Link, one person was excluded for wearing it for < 72 h (*n* = 36).

The comparison between the physical activity values estimated using the IPAQ‐S questionnaire and those obtained using accelerometry showed significant discrepancies in most of the variables analysed (Table 3).

**TABLE 3:** Comparison of daily physical activity levels estimated by accelerometry and the IPAQ‐S questionnaire, and correlation between methods in the subsample of 36 taxi driver*s*.

	Accelerometry	IPAQ‐S	Mean difference	95% CI		*r*
				Lower	Upper	
Inactivity (min/day)^a^	715.47 ± 146.94	675.81 ± 342.57	39.67 ± 60.90	−83.96	163.30	−0.01
LPA (min/day)^b^	21.26 ± 8.23	81.67 ± 114.56	−60.41 ± 19.26**	−99.50	−21.32	−0.12
MPA (min/day)	32.01 ± 67.18	78.89 ± 77.99	−46.88 ± 19.23*	−85.92	−7.84	0.31
VPA (min/day)	10.37 ± 56.96	66.81 ± 92.95	−56.44 ± 16.33**	−89.58	−23.30	0.24
MVPA (min/day)	42.37 ± 31.21	145.69 ± 140.22	−103.32 ± 22.33**	−148.66	−57.99	0.47**
TPA (min/day)	63.63 ± 34.21	227.36 ± 211.81	−163.73 ± 34.76**	−234.29	−93.17	0.33*
TAP MET‐min/week	1967.74 ± 1750.90	4483.44 ± 4544.44	−2515.70 ± 591.10**	−3715.70	−1315.70	0.41*

Abbreviations: CI, Confidence Interval; IPAQ‐S, International Physical Activity Questionnaire—Short Form; LPA, Light Physical Activity; MET, Metabolic Equivalent of Task; MPA, Moderate Physical Activity; MVPA, Moderate‐to‐Vigorous Physical Activity; *r*, Spearman correlation coefficient between accelerometer and IPAQ‐S variables; SD, Standard Deviation; TAP, Total Activity‐Related Physical Activity; TPA, Total Physical Activity; VPA, Vigorous Physical Activity.

^a^ For the IPAQ‐S, the variable compared was “Sedentary time”.

^b^ For the IPAQ‐S, the variable compared was walking time.

Statistical significance indicated by *p* < 0.05 (*) and *p* < 0.01 (**).

Participants tended to report higher time spent on LPA, MPA, VPA, MVPA and TPA, when reported by IPAQ‐S as presented in Table 3. The mean difference between IPAQ‐S and accelerometry for TPA was 163.73 min/day (95% CI 93.17 to 234.29; *p* < 0.01), and for MVPA it was 103.32 min/day (95% CI 57.99 to 148.66; *p* < 0.01). The only category in which no statistically significant differences were found between methods was inactivity time.

Regarding the correlation between the two methods, low to moderate Spearman correlation coefficients were observed. The highest correlation was obtained for MVPA (*r* = 0.47; *p* < 0.01), followed by TAP MET‐min/week (*r* = 0.41; *p* < 0.05) and TPA (*r* = 0.33; *p* < 0.05). In contrast, the correlation was very low or even inverse for LPA (*r* = −0.12) and null for inactivity (*r* = −0.01), suggesting a lower accuracy of the IPAQ‐S questionnaire in estimating low‐intensity activities or sedentary behaviours.

As shown in Table 4, a binomial logistic regression analysis was performed to identify sociodemographic variables with MSDs in different anatomical locations.

**TABLE 4:** Results of the binomial logistic regression analysis for the association between sociodemographic variables and the presence of musculoskeletal pain in different body locations (*n* = 170).

Variables (references)	Neck	Shoulder	Forearm	Wrists or hands	Upper back	Lower back	Hips or legs	Knees	Ankles or feet
	OR (95% CI)	OR (95% CI)	OR (95% CI)	OR (95% CI)	OR (95% CI)	OR (95% CI)	OR (95% CI)	OR (95% CI)	OR (95% CI)
Age (continuous)	0.98 (0.96–1.01)	1.02 (0.98–1.10)	1.02 (0.97–1.06)	1.02 (0.97–1.06)	0.96 (0.92–0.99)*	0.97 (0.94–1.00)	1.03 (0.99–1.07)	1.02 (0.99–1.06)	0.99 (0.96–1.04)
Gender (male)	3.65 (1.43–9.28)**	1.45 (0.55–3.84)	2.13 (0.63–7.17)	2.55 (0.89–7.31)	3.17 (1.14–8.77)*	3.53 (1.40–8.86)**	1.15 (0.36–3.69)	0.81 (0.26–2.55)	2.55 (0.89–7.31)
BMI (continuous)	1.03 (0.96–1.09)	1.04 (0.98–1.11)	1.02 (0.94–1.12)	1.01 (0.94–1.10)	1.02 (0.94–1.10)	1.03 (0.97–1.10)	1.07 (0.99–1.15)	1.09 (1.02–1.18)*	1.04 (0.96–1.12)
Hours of work per day (≤ 4 h)	4.10 (1.18–14.22)*	2.34 (0.68–8.08)	2.70 (0.35–20.53)	1.86 (0.42–8.12)	1.95 (0.45–8.51)	2.43 (0.81–7.34)	2.13 (0.49–9.33)	0.96 (80.35–2.67)	0.81 (0.27–2.46)

Abbreviations: BMI, Body Mass Index; CI, Confidence Interval; OR, Odds Ratio.

Statistical significance indicated by *p* < 0.05 (*) and *p* < 0.01 (**).

Regarding sociodemographic variables, age showed a statistically significant association with upper back pain, demonstrating a protective effect (OR of 0.96, *p* < 0.05), suggesting that the likelihood of experiencing pain in this region decreases with increasing age.

Female gender was significantly associated with a higher probability of pain in several body regions: neck (OR = 3.65, *p* < 0.01), upper back (OR = 3.17, *p* < 0.05) and lower back (OR = 3.53, *p* < 0.01).

BMI was positively associated with knee pain (OR 1.09, *p* < 0.05), indicating an increase in the likelihood of pain in the knee as BMI increased.

With regard to working hours, working more than 4 h a day was associated with a higher risk of neck pain (OR of 4.10, *p* < 0.05).

As shown in Table 5, no statistically significant associations were found between MSDs and any of the physical activity or sedentary behaviour variables IPAQ‐S classification (Inactive, Minimally Active and HEPA active), adherence to WHO guidelines (yes/no), or sedentary behaviour (above or below 650 daily minutes) according to IPAQ‐S or accelerometry. A trend was observed for shoulder pain (*p* = 0.052), with a higher prevalence among participants with ≥ 650 min/day of sitting time, although this association did not reach statistical significance.

**TABLE 5:** Percentage of participants with MSD by body region according to variables related to aerobic physical activity and sedentary lifestyl*e*.

	Neck	Shoulder	Forearm	Wrists or hands	Upper back	Lower back	Hips or legs	Knees	Ankles or feet
IPAQ‐S (*n* = 170)									
WHO Recommendation									
No	36 (37.11)	27 (27.84)	10 (10.31)	18 (18.56)	12 (12.37)	28 (28.87)	18 (18.56)	21 (21.65)	17 (17.53)
Yes	26 (35.62)	16 (21.92)	8 (10.96)	7 (9.59)	14 (19.18)	28 (38.36)	10 (13.70)	15 (20.55)	8 (10.96)
*p* value	0.84	0.38	0.89	0.10	0.22	0.19	0.40	0.86	0.23
IPAQ‐S classification									
Inactive	9 (39.13)	8 (34.78)	2 (8.70)	3 (13.04)	3 (13.04)	9 (39.13)	7 (30.43)	3 (13.04)	5 (21.74)
Minimally active	20 (35.09)	14 (24.56)	7 (12.28)	13 (22.81)	9 (15.79)	17 (29.82)	8 (14.04)	15 (26.32)	6 (10.53)
HEPA active	33 (36.67)	21 (23.33)	9 (10.00)	9 (10.00)	14 (15.56)	30 (33.33)	13 (14.44)	18 (20.00)	14 (15.56)
*p* value	0.94	0.52	0.86	0.10	0.94	0.72	0.15	0.40	0.42
Sedentary									
< 650 min/day	36 (38.71)	29 (31.18)	12 (12.90)	16 (17.20)	16 (17.20)	31 (33.33)	15 (16.13)	23 (24.73)	15 (16.13)
≥ 650 min/day	26 (33.77)	14 (18.18)	6 (7.79)	9 (11.69)	10 (12.99)	25 (32.47)	13 (16.88)	13 (16.88)	10 (12.99)
*p* value	0.51	0.052	0.28	0.31	0.45	0.91	0.90	0.21	0.57
Accelerometry (*n* = 36)									
WHO Recommendation									
No	6 (60.00)	4 (40.00)	0 (0.00)	1 (10.00)	4 (40.00)	7 (70.00)	2 (20.00)	2 (20.00)	0 (0.00)
Yes	8 (30.77)	4 (15.38)	1 (3.85)	1 (3.85)	5 (19.23)	11 (42.31)	1 (3.85)	3 (11.54)	1 (3.85)
*p* value	0.11	0.11	0.53	0.47	0.20	0.14	0.12	0.51	0.53
Sedentary									
< 650 min/day	5 (41.67)	4 (33.33)	0 (0.00)	0 (0.00)	2 (16.67)	5 (41.67)	0 (0.00)	3 (25.00)	1 (8.33)
≥ 650 min/day	9 (37.50)	4 (16.67)	1 (4.17)	2 (8.33)	7 (29.17)	13 (54.17)	3 (12.50)	2 (8.33)	0 (0.00)
*p* value	0.81	0.26	0.47	0.30	0.41	0.48	0.20	0.17	0.15

*Note:* Values represent absolute frequency and percentage (%) of participants with musculoskeletal disorders (MSDs) in each body region, stratified by physical activity level and sedentary behaviour, according to IPAQ‐S (International Physical Activity Questionnaire—Short Form) and accelerometry. WHO guidelines refer to achieving ≥ 150 min/week of moderate‐intensity or ≥ 75 min/week of vigorous‐intensity physical activity, or an equivalent combination.

Abbreviation: HEPA, Health‐Enhancing Physical Activity.

Statistical significance assessed by chi‐square. Significant *p* values are indicated by *p* < 0.05 (*) and *p* < 0.01 (**).

## 4 Discussion

This article aimed to describe the levels of physical activity and sedentary behaviours of taxi drivers using objective (accelerometry) and self‐reported (IPAQ‐S) tools, to assess their association with the prevalence of MSDs, and to analyse the discrepancies between the two measurement methods.

The results showed a high prevalence of MSDs in this population. In total, 68.8% of taxi drivers reported pain in at least one body region during the previous 7 days, with the most affected areas being the neck (36.5%) and lower back (32.9%). Significant percentages were also observed in the shoulders (25.3%), knees (21.2%) and hips (16.5%). These figures are consistent with previous studies of professional drivers, where the cervical and lumbar regions are usually the most affected due to prolonged static postures, vehicle vibration and limited seat ergonomics (Joseph et al. 2020; Rezaei et al. 2024).

Beyond these mechanical explanations, the high 7‐day symptom prevalence may also reflect the interaction of prolonged constrained sitting with work‐organisation factors (e.g., long shifts, limited opportunities for posture variation, time pressure, and psychosocial stress). This is particularly relevant given that a 7‐day recall window may be sensitive to short‐term workload peaks and recovery patterns.

Accelerometry allowed the actual levels of physical activity to be compared with the self‐reported data from the IPAQ‐S questionnaire. A significant overestimation was evident in virtually all IPAQ‐S physical activity categories, especially in MVPA, with mean values of 145.70 ± 140.20 min/day according to the questionnaire, compared to 42.37 ± 31.21 min/day recorded by accelerometry. A similar discrepancy was observed in TPA, with 227.40 ± 211.80 min/day self‐reported versus 63.63 ± 34.21 min/day.

These discrepancies between self‐reported and objectively measured data are consistent with what has been reported in the scientific literature. Previous studies in working populations—such as administrative staff, healthcare personnel, and professional drivers—have documented that the IPAQ‐S tends to overestimate physical activity compared with accelerometry, with margins ranging from 36% to 173% depending on the intensity of the activity and the type of workday (Lee et al. 2011; Nelson et al. 2019). This divergence can be attributed to various factors, such as social desirability bias, recall errors, or subjective perception of physical effort (Sánchez‐Lastra and Martínez‐Lemos 2018). However, this may have important implications when analysing the true relationship between physical activity and pain in these populations.

Based on the accelerometry data, due to the objectivity of the test, in our sample, the inactivity time measured by accelerometry exceeded 700 min per day (11 h and 40 min), which is comparable to that reported in other studies with transport and administrative sector workers (Bailey 2021; Murray et al. 2019; Prince et al. 2019; Rezaei et al. 2024; Varela‐Mato et al. 2016). This level of sedentary behaviour represents a significant risk factor for the development of non‐communicable diseases and MSDs. According to Park et al. (2020), compliance with physical activity recommendations does not necessarily offset the risks associated with prolonged sedentary behaviour, which is an independent risk factor for mortality (Park et al. 2020). In the case of taxi drivers, this distinction is particularly relevant, as most may overestimate their level of physical activity, without realising that spending more than 8 h a day sitting down carries a substantial risk to musculoskeletal and metabolic health.

One aspect that deserves attention is the effect of gender. Despite the small number of women in the sample (12.9%), being female was significantly associated with a higher probability of pain in the neck, upper back and lower back. These findings, although preliminary, are consistent with studies in other contexts that have shown a higher prevalence of MSDs in female drivers (Berrones‐Sanz and Araiza‐Diaz 2019; Szeto and Lam 2007), possibly due to a complex interaction of biological, psychosocial, structural and dual exposure factors (work‐home demands; Fillingim et al. 2009; Punnett and Herbert 2000). However, given the small proportion of women and the region‐specific modelling approach, these sex‐related estimates may be statistically imprecise and should be interpreted as exploratory.

Additionally, BMI was significantly associated with knee pain. While previous studies have typically linked elevated BMI to lower back pain (Shiri et al. 2010), our findings suggest a stronger association with the knees in this population. The mechanistic basis for this relationship is likely multifactorial. Although mechanical overload is a well‐established pathway (Muehleman et al. 2010) in a highly sedentary population, the contribution of sustained joint loading from prolonged sitting and suboptimal posture may be more relevant than repetitive weight‐bearing activities. Furthermore, excess adiposity is associated with chronic low‐grade systemic inflammation, which has been implicated in the progression of knee osteoarthritis and other musculoskeletal disorders (Vincent et al. 2013). This inflammatory component, combined with reduced muscle strength from physical inactivity, may exacerbate joint vulnerability and pain. Previous work has shown that obesity heightens the risk and severity of musculoskeletal pain across several anatomical sites (Mendonça et al. 2020; Nilsen et al. 2011), and such effects may be amplified in individuals with prolonged sedentary time and low physical activity levels.

Although our study did not find a significant association between PA levels and MSDs, this finding should not be interpreted as an absence of a relationship in general terms. One possible explanation is the existence of a ceiling effect or exposure threshold, whereby the benefits of physical activity are attenuated in populations subjected to extreme sedentary lifestyles, as confirmed by Ribas et al. (2020) in their study of administrative workers (Ribas et al. 2020). In such cases, even adequate levels of PA may not be sufficient to counteract the accumulated risk of MSDs. This would explain why in our sample, neither self‐reported nor objectively measured levels showed a clear protective effect. In Australia, a study of more than 220,000 adults estimated that sedentary behaviour could be responsible for 6.9% of deaths in the population. This highlights the seriousness of the problem and suggests that reducing sitting time should be a priority objective alongside promoting PA, even when seeking to prevent MSDs (van der Ploeg 2012).

More broadly, the absence of significant associations between physical activity and MSDs, and between sedentary time and MSDs, may also reflect methodological and contextual factors. First, non‐differential misclassification of PA due to systematic self‐report overestimation (and the limited accelerometry subsample) can bias associations toward the null. Second, the occupational context of taxi driving entails very high—and potentially relatively homogeneous—sedentary exposure, which may restrict variability and reduce detectable associations. Third, reverse causality is plausible in a cross‐sectional design, as musculoskeletal pain may influence activity patterns and break behaviour. Fourth, total sedentary time may not capture the specific biomechanical exposures most directly linked to regional pain (e.g., seat ergonomics, vibration exposure, driving hours, and opportunities for micro‐breaks), and residual confounding by these unmeasured factors may obscure associations. Finally, some region‐specific analyses may have limited statistical power and precision, yielding imprecise estimates. Taken together, these issues suggest that the null associations observed in this sample should be interpreted cautiously and warrant investigation in adequately powered longitudinal studies incorporating detailed occupational exposure metrics and objective monitoring.

On the other hand, it is worth reflecting on the validity of using the IPAQ‐S questionnaire in taxi drivers. As we have shown, this instrument tends to significantly overestimate the time spent on physical activity compared with objective measurement. From a measurement perspective, combining objective monitoring with instruments tailored to the task structure of driving (e.g., capturing micro‐breaks, posture changes, and domain‐specific activity) may improve exposure assessment and reduce misclassification in future studies.

Returning to sedentary time, the absence of an association with MSDs in our data should not be interpreted as definitive either. Uncontrolled variables (seat ergonomics, the presence of active breaks, stress levels or rest) may modulate this relationship (Straker et al. 2016). In addition, musculoskeletal pain may depend more on exposure to vibrations or uncomfortable postures than on overall sedentary behaviour itself, although both aspects are interrelated (Lis et al. 2007).

Finally, although this study did not find a protective effect of PA on MSDs, its importance in other key dimensions of health should not be minimised. Physical activity is widely associated with cardiovascular, metabolic, psychological and functional benefits (Oral et al. 2024; Villamil‐Parra and Moscoso‐Loaiza 2024), aspects that were not evaluated here but should be taken into account when designing public health strategies for this group.

Psychosocial determinants may also shape symptom experience and health behaviours in taxi drivers. Our prior Spain–Chile study linked stress, anxiety and depression to poorer quality of life and suggested occupational balance as a protective factor, supporting integrated biopsychosocial approaches alongside ergonomic and organisational strategies (Marín‐Berges et al. 2025).

Taken together, the main contribution of this study is descriptive and methodological: it documents a high 7‐day MSD burden alongside marked sedentary exposure and substantial overestimation of PA by self‐report in a highly sedentary occupational group. The absence of clear associations should be interpreted considering measurement limitations, cross‐sectional design, and potential residual confounding. These findings refine hypotheses and can inform the design of future studies (larger objective monitoring, inclusion of detailed occupational exposure metrics, and longitudinal follow‐up) rather than supporting causal inference.

### 4.1 Strengths and Limitations

One of the main strengths of this study is its mixed approach to assessing physical activity by combining self‐reporting tools (IPAQ‐S) with objective measurement using accelerometry. This approach made it possible to compare subjective perceptions with actual movement data, providing a more accurate picture of physical behaviour in a working population. In addition, the study covered a large sample of taxi drivers from two different geographical contexts (Spain and Chile), which contributes to the diversity and applicability of the findings in other urban environments.

Likewise, the use of the SNQ allowed for a detailed and validated assessment of musculoskeletal disorders by body region, which strengthens the quality of the data collected on musculoskeletal health.

However, this study has some limitations. First, accelerometry was only used with a subsample of 36 participants, which restricts the generalisation of the objective results to the entire sample.

Furthermore, although the use of an accelerometer on the wrist of the non‐dominant hand is an accepted practice for professional drivers (Gilson et al. 2021), it may not fully reflect actual energy expenditure in certain activities, especially in drivers where upper limb movement may be restricted or influenced by driving.

Another limitation is the cross‐sectional nature of the design, which prevents the establishment of causal relationships between physical activity and MSDs.

## 5 Conclusion

Taxi drivers exhibited a high prevalence of 7‐day MSDs, particularly in the neck and lower back, with a higher reported burden among women. Although more than half of the participants were classified as sufficiently active based on questionnaire data, accelerometer data (available in a subsample from Zaragoza) indicated very high sedentary time and substantially lower physical activity, suggesting overestimation in self‐reports. Associations between physical activity and 7‐day MSDs were weak, and sedentary time was not clearly associated with MSD prevalence in this sample. These findings should be interpreted cautiously given the cross‐sectional design, reliance on self‐report measures, and the limited proportion of participants with accelerometer data.

Overall, the results highlight a high MSD burden alongside marked sedentary exposure in taxi drivers and underscore the importance of improving measurement in highly sedentary occupational groups. Future research should include larger samples with objective monitoring across settings (e.g., Valparaíso as well as Zaragoza), incorporate detailed occupational exposure measures (e.g., ergonomics and work organisation), and evaluate workplace strategies (e.g., ergonomic optimisation and feasible break routines) using adequately powered and preferably longitudinal designs.

## Author Contributions

Marta Marín‐Berges led the statistical analysis, contributed to data collection, interpretation of results, and drafted the initial manuscript. Pablo A. Lizana contributed to study conception, data collection, and interpretation of results. Isabel Iguacel contributed to the study design, interpretation of results, and critically revised the manuscript for important intellectual content. Marcos Echevarría‐Polo contributed to statistical analysis, interpretation of results, and manuscript revision. Valentina Marroquín‐Pinochet contributed data collection, and interpretation of results. Constanza Rivas‐Sanhueza contributed to data collection and interpretation of results. German Vicente‐Rodríguez contributed to study conception, interpretation of results, and manuscript revision. Alejandro Gómez‐Bruton contributed to study conception, statistical analysis, interpretation of results, and manuscript revision. All authors contributed to the study design and interpretation of data, critically reviewed and approved the final version of the manuscript, and agree to be accountable for all aspects of the work.

## Funding

The authors have nothing to report.

## Ethics Statement

This study was approved by the Research Ethics Committee of the Autonomous Community of Aragón (PI22‐382) and by the Bioethics Committee of the Pontificia Universidad Católica de Valparaíso (n◦BIOEPUCV‐H 633–2023). All participants provided voluntary consent by signing the informed consent form after being fully informed about the study's objectives. They were also made aware of their right to withdraw from the study at any point without consequence.

## Conflicts of Interest

The authors declare no conflicts of interest.

## Data Availability

The datasets generated and/or analysed during the current study are not publicly available due to participant confidentiality and the lack of consent for data sharing.
